# Massas Pericárdicas: Apresentação Rara de Pericardite Tuberculosa, Documentada em Ecocardiografia 3D

**DOI:** 10.36660/abc.20190876

**Published:** 2021-02-02

**Authors:** Alex dos Santos Felix, Viviane Belidio Pinheiro da Fonseca, Rodrigo Coelho Segalote, Larissa Franco de Andrade, Deborah Louize da Rocha Vianna Palmieri, Ana Paula dos Reis Velloso Siciliano

**Affiliations:** 1 Instituto Nacional de Cardiologia Rio de JaneiroRJ Brasil Instituto Nacional de Cardiologia, Rio de Janeiro, RJ – Brasil

**Keywords:** Neoplasias Cardíacas/cirurgia, Pericardite Tuberculosa/fisiopatologia, Tamponamento Cardíaco/cirurgia, Ecocardiografia Tridimensional/métodos, Diagnóstico por Imagem/métodos

## Pontos de Aprendizagem

- Massas pericárdicas são frequentemente causadas por tumores metastáticos ou primários, mas podem, em casos raros, ser causadas por doenças inflamatórias, infecciosas ou granulomatosas, como a pericardite tuberculosa.
- Em casos graves, com extenso derrame e comprometimento hemodinâmico (tamponamento pericárdico), a drenagem é obrigatória e o manejo padrão-ouro inclui pericardiotomia com drenagem completa e ressecção da massa para um melhor resultado terapêutico, para contribuir para o diagnóstico e evitar o reacúmulo de líquido.
- Técnicas de imagem multimodal e ecocardiografia 3D para retratar melhor os detalhes da massa, sua fixação às estruturas adjacentes, bem como para avaliar outras estruturas torácicas e mediastinais, são métodos valiosos para esclarecer a etiologia correta e excluir diagnósticos diferenciais.

## Introdução

A pericardite é uma manifestação rara da tuberculose (TB) que, apesar de ocorrer em apenas 2% dos casos, ^1^ é responsável por aproximadamente 70% de todos os casos de grande derrame pericárdico e a maioria dos casos de pericardite constritiva em países em desenvolvimento. Massas pericárdicas são apresentações muito raras da TB e podem ser confundidas com tumores pericárdicos primários ou metastáticos. Dito isso, deve haver uma avaliação imediata e cuidadosa para se descartar malignidade subjacente.

## Relato de Caso

Paciente do sexo masculino, 29 anos, admitido no Pronto Socorro com ortopneia e edema periférico, referindo dispneia progressiva nas últimas duas semanas, após febre baixa prolongada, artralgia e emagrecimento (16 kg) nos últimos cinco meses. O paciente não tinha histórico de doenças cardiovasculares ou pulmonares. Exame de chegada mostrou taquicardia, sinais de desconforto respiratório, pulso regular e bulhas hipofonéticas à ausculta cardíaca. Hepatomegalia e edema de membros inferiores 2+/4+ foram observados, sendo que todos os outros aspectos clínicos estavam normais.

### Investigação

Eletrocardiograma mostrou complexos QRS com baixa voltagem e taquicardia. Nos exames laboratoriais, destacava-se aumento da proteína C-reativa (9,72 mg/L). O resultado da contagem de leucócitos foi normal e as hemoculturas foram todas negativas.

**Figura 1:**
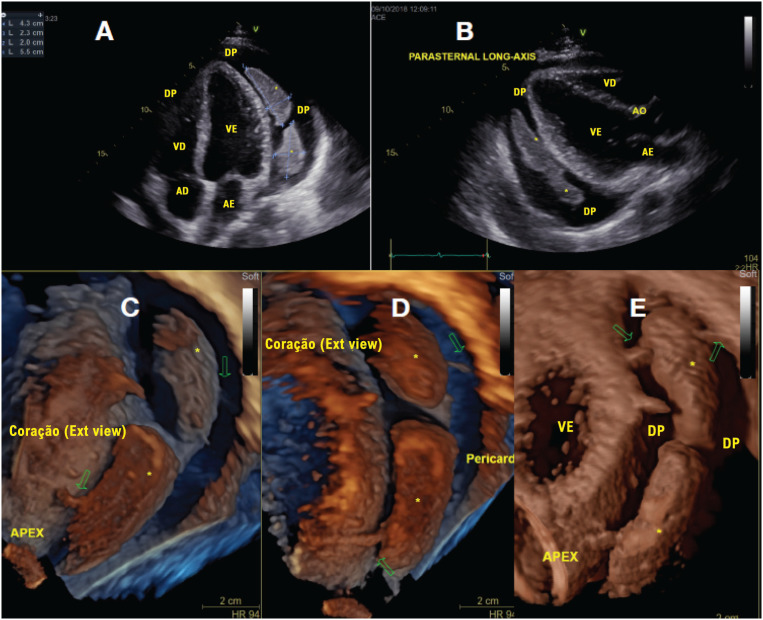
Ecocardiograma transtorácico (ETT) demonstrando volumoso derrame pericárdico com massas intrapericárdicas (*). Ao ECO2D são bem visualizadas duas massas discoides medindo 5.5×2.0cm e 4.3×2.3cm, evidenciáveis a partir das projeções apical 4 câmaras (A) e paraesternal (B) em uma posição póstero-lateral ao coração, em meio ao grande derrame pericárdico. Imagens renderizadas de ECO3D foram obtidas a partir do pós-processamento de “datasets” adquiridos por ETT3D. Podemos evidenciar em C, D e E, em cortes 3D oblíquos, os detalhes morfológicos das massas, com traves fibrinoides que as aderem ao pericárdio parietal e visceral (setas), flutuando dentro do líquido pericárdico e sem invasão de tecidos circunjacentes. VE: ventrículo esquerdo; VD: ventrículo direito; AE: átrio esquerdo; AD: átrio direito; DP: derrame pericárdico.

A suspeita era de tamponamento pericárdico e foi realizado ecocardiograma transtorácico (ETT). Um grande derrame pericárdico foi detectado, com aumento da variação respiratória no pico de velocidade das ondas E no influxo mitral (>25%) e tricúspide (>50%), veia cava inferior (VCI) dilatada com aumento do reverso expiratório do fluxo de veia hepática, apontando restrição diastólica. Além disso, a ecocardiografia mostrou pericárdio espessado com irregularidades superficiais e duas grandes massas intrapericárdicas de contornos regulares, medindo 5,5×2,0 cm e 4,3×2,3 cm, interligadas por uma ponte de tecido, fixadas às camadas visceral e parietal do pericárdio por traves fibrinosas, flutuando dentro do líquido pericárdico e sem invadir os tecidos circunvizinhos, melhor detalhadas pela análise 3D ( Figura 1 , Vídeos 1–2). A função sistólica biventricular estava normal. Uma tomografia computadorizada (TC) torácica mostrou linfonodomegalia mediastinal e ausência de lesões no parênquima pulmonar.

**Vídeo 1:** Ecocardiograma transtorácico nas projeções parasternal e apical 4 câmaras, demonstrando duas massas intrapericárdicas discoides flutuando dentro de derrame pericárdico volumoso. VE: ventrículo esquerdo; VD: ventrículo direito; AE: átrio esquerdo; AD: átrio direito; DP: derrame pericárdico. Acesse o vídeo pelo link: http://abccardiol.org/supplementary-material/2021/11601/2019-0876-video1.mp4

**Vídeo 2:** Imagens 3D renderizadas pós-processadas a partir de um dataset adquirido por ecocardiograma transtorácico, demonstrando por cortes oblíquos um pericárdio espessado com irregularidades de superfície e duas grandes massas intrapericárdicas com contornos regulares, interconectadas por uma ponte de tecido entre si, aderidas às laminas pericárdicas parietal e visceral por traves fibrosas, flutuando no interior de volumoso derrame pericárdico, sem invasão de tecidos circunjacentes. VE: ventrículo esquerdo; VD: ventrículo direito; AE: átrio esquerdo; AD: átrio direito; DP: derrame pericárdico. Acesse o vídeo pelo link: http://abccardiol.org/supplementary-material/2021/11601/2019-0876-video2.mp4

### Tratamento e resultado

**Figura 2:**
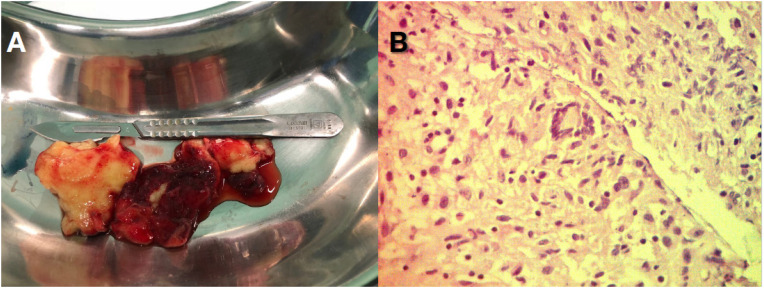
Análise histopatológica das massas intrapericárdicas. Em A evidenciamos o aspecto macroscópico das duas massas, que tem formato discoides e se constituem por tecido lobulado amarelado e gelatinoso. No exame histopatológico (B) com coloração de hematoxilina-eosina (40x), há um padrão de inflamação granulomatosa crônica com necrose, característico de granuloma tuberculoso.

Foi iniciado tratamento empírico para TB com rifampicina (R), isoniazida (H), pirazinamida (Z) e etambutol (E) por via oral e, devido à instabilidade hemodinâmica, o paciente foi submetido a pericardiotomia de urgência. Foi realizada drenagem de secreção serossanguinolenta (600 ml) e as massas foram completamente excisadas ( Figura 2-A , Vídeo 3). As massas tinham formato discoide e eram macroscopicamente compostas por um tecido mole amarelado lobulado. Uma amostra da massa foi submetida a exame de congelação no intraoperatório, descartando-se malignidade. A histopatologia revelou um padrão de inflamação granulomatosa crônica com necrose, compatível com TB ( Figura 2-B ).

**Vídeo 3:** Imagens do intraoperatório (visão do cirurgião). O paciente foi submetido a pericardiotomia de urgência. Um grande volume de fluido sero-sanguinolento sob alta pressão foi drenado (600ml), e realizada a excisão completa das traves fibrosas e das massas. Acesse o vídeo pelo link: http://abccardiol.org/supplementary-material/2021/11601/2019-0876-video3.mp4

O paciente teve alta hospitalar com prescrição de RHZE via oral por dois meses, seguido de RH por mais quatro meses, sem intercorrências ou recidivas e com boa evolução.

## Discussão

A pericardite tuberculosa é uma apresentação incomum da TB, ocorrendo em apenas 2% dos casos, ^1^ Geralmente é causada pela disseminação retrógrada de bacilos do *Mycobacterium tuberculosis* a partir dos linfonodos peritraqueais, peribrônquicos ou mediastinais, ou por disseminação hematogênica a partir da infecção tuberculosa primária. ^2^

Massas pericárdicas são relativamente raras, na maioria dos casos sendo causadas por malignidades, sendo o envolvimento metastático do pericárdio mais frequente do que tumores primários, muitas vezes com um prognóstico ruim. ^3^ Doenças inflamatórias e infecciosas são mais raramente relatadas como causadoras de massas pericárdicas na literatura, com poucos relatos de equinococose cardíaca, ^4^ artrite reumatoide, ^5^ pseudotumores inflamatórios ^6^ e pericardite tuberculosa. ^7^ A apresentação varia e os pacientes costumam ser assintomáticos, com envolvimento pericárdico detectado apenas na autópsia ou como achado incidental durante exames de imagem torácica. Alguns pacientes, entretanto, podem desenvolver sintomas progressivos de congestão venosa devido à evolução de derrame pericárdico (restrição diastólica) ou constrição, apresentando dispneia, ortopneia e edema periférico. ^8^ Em casos graves, pode ocorrer tamponamento cardíaco e choque cardiogênico, o que requer intervenção urgente para drenagem de fluidos e/ou pericardiectomia.

Neste relato, descrevemos um caso de pericardite tuberculosa com extenso derrame pericárdico e duas grandes massas discoides flutuando dentro do líquido pericárdico e com fixações nas camadas parietal e visceral do pericárdio, bem ilustrado por imagens do ETT 3D. As massas pericárdicas causadas pela TB são muito raras e apenas alguns casos estão relatados na literatura médica até o momento. ^7^ ^,^ ^9^ ^–^ ^12^ Há relato de pelo menos cinco casos semelhantes de pericardite por TB associada a massas pericárdicas, quatro em pacientes pediátricos e um em um homem de 19 anos. ^9^

A fisiopatologia dessas massas ainda é pouco compreendida e considerada resultado de um conglomerado de hemácias e materiais proteicos no líquido pericárdico, secundário à pericardite por TB ^7^ . Em nosso caso, os achados histológicos foram um pouco diferentes desta descrição, pois encontramos um processo inflamatório granulomatoso crônico, com aspecto típico de um granuloma de TB. Nosso paciente não apresentava pneumonia tuberculosa clínica e não correspondia aos critérios diagnósticos clássicos de pericardite tuberculosa, pois não foram encontrados bacilos no líquido pericárdico ou nas amostras histológicas obtidas do pericárdio excisado e das massas. No entanto, o achado de um granuloma caseoso típico de TB ao exame microscópico estabeleceu o diagnóstico final.

## Conclusão

As massas pericárdicas são uma apresentação rara de pericardite tuberculosa, com poucos casos relatados até o momento. O pronto diagnóstico e tratamento precoce são importantes para um bom desfecho, assim como as técnicas de imagem multimodais são fundamentais para o diagnóstico diferencial de outras causas de massas cardíacas, como tumores. Este caso ilustra o valor adicional das imagens multimodais e da ecocardiografia 3D para um diagnóstico preciso, fornecendo dados importantes para a tomada de decisão e uma estratégia de tratamento eficaz.

## References

[1] 1. Fowler NO. Tuberculous pericarditis. JAMA. 1991;266(1):99–103. 2046135

[2] 2. Commerford PJ, Strang JIG. Tuberculous pericarditis. In: Coovadia HM, Benatar SR, eds. A Century of Tuberculosis: South African Perspectives. Cape Town: Oxford University Press; 1991.p.123–36

[3] 3. Luk A, Ahn E, Vaideeswar P, Butany JW. Pericardial tumors. Semin Diagn Pathol. 2008;25(1):47-53. 10.1053/j.semdp.2007.12.001 18350922

[4] 4. Oliver JM, Sotillo JF, Domínguez FJ, Lopes de Sá E, Calvo L, Salvador A, et al. Two-dimensional echocardiographic features of echinococcosis of the heart and great blood vessels. Clinical and surgical implications. Circulation. 1988 Aug;78(2):327-37. 10.1161/01.cir.78.2.327 3396169

[5] 5. Cañas F, León JDLP, Gomez JE, Cañas CA. A giant fibrinoid pericardial mass in a patient with rheumatoid arthritis: a case report. Eur Heart. Case Reports.2019;3(2):1-5. 10.1093/ehjcr/ytz061 PMC6601188 31449627

[6] 6. Blanco M, Fulquet E, Laguna G, Martinez G, Sevilla T, Di Stefano S, et al. Cardiac Inflammatory Myofibroblastic Tumor in a Young Male Patient With Myopericarditis. Circulation. 2015;132(25):e386–e387. 10.1161/CIRCULATIONAHA.115.018671 26700010

[7] 7. Yoon SA, Hahn YS, Hong JM, Lee OJ, Han HS. Tuberculous pericarditis presenting as multiple free floating masses in pericardial effusion. J Korean Med Sci. 2012;27(3):325-8. 10.3346/jkms.2012.27.3.325 PMC3286783 22379347

[8] 8. Mady C, Fernandes F, Ohki F. Tumores do pericárdio. Rev Soc Cardiol Estado de São Paulo. 2011;21(1):38-41.

[9] 9. Li C, Zhao Q, Wu X, Yu J. Tuberculous pericarditis mimicking multiple tumors in pericardial effusion. J Int Med Res. 2019;47(5):2262-8. 10.1177/0300060519834454 PMC6567750 30898056

[10] 10. Lin JH, Chen SJ, Wu MH, Lee PI, Chang CI. Fibrinofibrous pericarditis mimicking a pericardial tumor. J Formos Med Assoc. 2000;99(1):59-61. 10743349

[11] 11. Massoure PL, Boddaert G, Caumes JL, Gaillard PE, Lions C, Grassin F. Porridge-like tuberculous cardiac tamponade: treatment difficulties in the Horn of Africa. Gen Thorac Cardiovasc Surg. 2010;58(6):276-8. 10.1007/s11748-009-0525-y 20549456

[12] 12. Garg A, Rajamouli DS, Eknath JU, Vasant KJ. Tuberculous pseudotumor of the pericardium: a case report. Indian J Thorac Cardiovasc Surg. 2013;29:262-4.

