# Supplementary material for: Massas Pericárdicas: Apresentação Rara de Pericardite Tuberculosa, Documentada em Ecocardiografia 3D
Source: Arq Bras Cardiol. 2021 Feb 2;116(2 Suppl 1):12–6. [Article in Portuguese] doi: 10.36660/abc.20190876 (PMC8118636; doi:10.36660/abc.20190876)
Supplement: Translation

# Translation: Pericardial Masses: A Rare Presentation of Tuberculous Pericarditis Documented by 3D Echocardiography

**Authors:** Alex dos Santos Felix, Viviane Belidio Pinheiro da Fonseca, Rodrigo Coelho Segalote, Larissa Franco de Andrade, Deborah Louize da Rocha Vianna Palmieri, Ana Paula dos Reis Velloso Siciliano

**Affiliations:** 1 Rio de JaneiroRJ Brazil Instituto Nacional de Cardiologia, Rio de Janeiro, RJ – Brazil

**Keywords:** Heart Neoplasms/surgery, Pericarditis, Tuberculous/physiopathology, Cardiac Tamponade/surgery, Echocardiography, Three-Dimensional/methods, Diagnosis, Imaging/methods

## Learning Points

- Pericardial masses are frequently caused by metastatic or primary tumors, but may be rarely caused by inflammatory, infectious or granulomatous diseases such as tuberculous pericarditis.
- In severe cases, with large effusion and hemodynamic impairment (pericardial tamponade), drainage is mandatory, and the gold-standard management includes pericardiotomy with complete drainage and mass resection to achieve a better therapeutic result, contribute to diagnosis and avoid fluid re-accumulation.
- Multimodality imaging and the use of 3D echocardiography to better depict details of the mass, its attachment to adjacent structures and to evaluate other thoracic and mediastinal structures is greatly valuable to clarify the correct etiology and exclude differential diagnoses.

## Introduction

Pericarditis is a rare manifestation of tuberculosis (TB) that, despite occurring in only 2% of cases, ^1^ is responsible for approximately 70% of all cases of large pericardial effusion and most cases of constrictive pericarditis in developing countries. Pericardial masses are very rare presentations of TB, and can be mistaken as primary or metastatic pericardial tumors. That being said, there should be a prompt and careful evaluation to rule out underlying malignancy.

## Case Report

A 29-year-old male was admitted to the Emergency Room with orthopnea and peripheral edema, reporting progressive dyspnea over the last two weeks, after protracted low fever, arthralgia, and weight loss (16 kg) over the last five months. The patient did not have a history of cardiovascular or pulmonary diseases. Primary examination showed tachycardia, signs of respiratory distress, regular pulse, and hypophonic heart sounds. Hepatomegaly and peripheral edema 2+/4+ were noted, and all other clinic aspects were normal.

### Investigation

Electrocardiography showed low QRS voltage and tachycardia. In laboratory tests, increased C-reactive protein (9.72 mg/L) stood out. Leukocyte count was normal and hemocultures were all negative.

The suspicion was pericardial tamponade, and a transthoracic echocardiogram (TTE) was performed. A large pericardial effusion was detected, with increased respiratory variation in peak E-waves velocity in mitral (>25%) and tricuspid inflow (>50%), dilated inferior vena cava (IVC) with increased hepatic vein expiratory flow reversal, pointing to diastolic restriction. Additionally, echocardiography showed a thickened pericardium with surface irregularities and two large intrapericardial masses with regular contours, measuring 5.5×2.0cm and 4.3×2.3cm, interconnected by a bridge of tissue, attached to visceral and parietal layers of the pericardium by fibrinous strands, floating inside the pericardial fluid and not invading surrounding tissues, better depicted by 3D analysis ( Figure 1 , Videos 1–2). Biventricular systolic function was normal. A thoracic computed tomography (CT) scan showed mediastinal lymph node enlargement and no lesions in the lung parenchyma.

**Figure 1:** Transthoracic echocardiogram (TTE), showing large pericardial efusion with two intrapericardial masses (*). Two discoid masses were detected by 2D TTE, measuring 5.5 × 2.0cm and 4.3 × 2.3cm in its major diameters, from apical (A) and parasternal views (B). Rendered 3D images were obtained by post processing of datasets obtained by 3DTTE. Oblique 3D views in C, D and E show morphologic details of the masses, which are attached to visceral and parietal pericardial layers by fibrinous strands (arrows), floating inside the pericardial fluid, without invasion of surrounding tissues. LV: Left ventricle; RV: Right ventricle; LA: Left atrium; RA: Right atrium; PE: Pericardial effusion.

**Video 1:** Transthoracic Echocardiogram from apical 4 chamber and parasternal views, showing two disc-shaped intrapericardial masses floating inside large pericardial effusion. LV: Left ventricle; RV: Right ventricle; LA: Left atrium; RA – Right atrium; PE: Pericardial effusion. Access the video at the link: http://abccardiol.org/supplementary-material/2021/11601/2019-0876-video1.mp4

**Video 2:** 3D rendered images from a dataset acquired from transthoracic echocardiogram, showing in oblique views a thickened pericardium with surface irregularities and two large intrapericardial masses with regular contours, interconnected by a bridge of tissue, attached to visceral and parietal layers of the pericardium by fibrinous strands, floating inside the pericardial fluid and not invading surrounding tissues. LV: Left ventricle; RV: Right ventricle; LA: Left atrium; RA: Right atrium; PE: Pericardial effusion. Access the video at the link: http://abccardiol.org/supplementary-material/2021/11601/2019-0876-video2.mp4

### Treatment and Outcome

Empirical treatment for TB with oral rifampin (R), isoniazid (H), pyrazinamide (Z), and ethambutol (E) was started and, because of hemodynamic instability, the patient underwent urgent pericardiotomy. Serosanguinous drainage (600 ml) was performed and the masses were completely excised ( Figure 2-A , Video 3). The masses were disc-shaped and macroscopically composed by a lobulated yellowish soft tissue. A specimen from the mass was submitted to intraoperative frozen section (IFS), ruling out malignancy. Histopathology revealed a pattern of chronic granulomatous inflammation with necrosis, consistent with TB ( Figure 2-B ).

**Figure 2:** Histopathologic analysis of the intrapericardial masses. Macroscopically (A) the masses were disc-shaped and constituted by a lobulated yellowish soft tissue. In B, histopathological analysis with hematoxylin/eosin stain (magnification 40x) shows a pattern of chronic granulomatous inflammation with necrosis, consistent with tuberculosis granuloma.

**Video 3:** Intraoperative images (surgical view). The patient underwent urgent pericardiotomy. A large volume of serosanguinous fluid under pressure was drained (600 ml), and complete excision of fibrinous strands and masses were performed. Access the video at the link: http://abccardiol.org/supplementary-material/2021/11601/2019-0876-video3.mp4

The patient was discharged from hospital on oral RHZE and prescription for two months, followed by RH for four more months, without any complications or relapses and showing good outcome.

## Discussion

Tuberculous pericarditis is an uncommon presentation of TB, occurring in only 2% of cases. ^1^ It is usually caused by retrograde spread of *Mycobacterium tuberculosis bacilli* from peritracheal, peribronchial or mediastinal lymph nodes, or by hematogenous spread from primary tuberculous infection. ^2^

Pericardial masses are relatively rare, mostly caused by malignancies, and the metastatic involvement of the pericardium is more frequent than primary tumors, often carrying a poor prognosis. ^3^ Inflammatory and infectious diseases are very rarely reported as causes of pericardial masses in the literature, with few reports of cardiac echinococcosis, ^4^ rheumatoid arthritis, ^5^ inflammatory pseudotumors, ^6^ and tuberculous pericarditis. ^7^ The presentation varies and patients are often asymptomatic, with pericardial involvement detected only at the autopsy or as an incidental finding during thoracic imaging tests. Some patients, however, may develop progressive symptoms of venous congestion due to evolution of pericardial effusion (diastolic restriction) or constriction, presenting with dyspnea, orthopnea, and peripheral edema. ^8^ In severe cases, cardiac tamponade and cardiogenic shock may occur, which requires urgent intervention for fluid drainage and/or pericardiectomy.

In this report, we described a case of tuberculous pericarditis with large pericardial effusion and two large discoid masses floating inside the pericardial fluid and with attachments to pericardial parietal and visceral layers, well depicted with 3D TTE images. Pericardial masses caused by TB are very rare, and only a few cases have been reported in the medical literature so far. ^7^ ^,^ ^9^ ^–^ ^12^ There are at least five similar cases of TB pericarditis associated to pericardial masses, four in pediatric patients and one in a 19-year-old man ^9^ .

The pathophysiology of these masses is still poorly understood and suggested to be the result of a conglomerate of red blood cells and protein materials inside the pericardial fluid, secondary to TB pericarditis. ^7^ In our case, the histologic findings were somewhat different from this description, as we found a chronic granulomatous inflammatory process, which is a typical aspect of a TB granuloma. Our patient did not present with clinical TB pneumonia, and did not match the classical diagnostic criteria for TB pericarditis, as *tubercle bacilli* were not found in the pericardial fluid or in the histological specimens obtained from the excised pericardium and from the masses. However, the finding of a typical TB caseating granuloma upon microscopical examination stablished the final diagnosis.

## Conclusion

Pericardial masses are a rare presentation of tuberculous pericarditis, with few cases reported so far. Timely diagnosis and early treatment are important for a good outcome, as well as multimodal imaging is fundamental for differential diagnosis with other sources of cardiac masses such as tumors. This case illustrates the additional value of multimodal imaging and the use of 3D echocardiography for an accurate diagnosis, thus providing important data for decision-making and an effective treatment strategy.
